# Effect of Biomass as Nucleating Agents on Crystallization Behavior of Polylactic Acid

**DOI:** 10.3390/polym14204305

**Published:** 2022-10-13

**Authors:** Kang Shi, Guoshuai Liu, Hui Sun, Biao Yang, Yunxuan Weng

**Affiliations:** 1 College of Chemistry and Materials Engineering, Beijing Technology and Business University, Beijing 100048, China; 2 Beijing Key Laboratory of Quality Evaluation Technology for Hygiene and Safety of Plastics, Beijing Technology and Business University, Beijing 100048, China

**Keywords:** polylactic acid, biomass, nucleating agent, crystallization

## Abstract

Polylactic acid (PLA) is one of the most productive biodegradable materials. Its bio-based source makes it truly carbon neutral. However, PLA is hard to crystallize as indicated by a low crystallization rate and a low crystallinity under conventional processing conditions, which limits its wider application. One of the most effective ways to enhance the crystallization ability of PLA is to add nucleating agents. In the context of increasing global environmental awareness and the decreasing reserves of traditional petroleum-based materials, biomass nucleating agents, compared with commonly used petroleum-based nucleating agents, have received widespread attention in recent years due to their abundance, biodegradability and renewability. This paper summarizes the research progress on biomass nucleating agents for regulating the crystallization behavior of polylactic acid. Examples of biomass nucleating agents include cellulose, hemicellulose, lignin, amino acid, cyclodextrins, starch, wood flour and natural plant fiber. Such green components from biomass for PLA are believed to be a promising solution for the development of a wholly green PLA-based system or composites.

## 1. Introduction

Polylactic acid (PLA) is widely used in fields such as biomedicine [1], packaging coatings [2,3], electronic appliances [4,5], 3D printing [6,7] and a wide range of other applications owing to its excellent biodegradability and mechanical properties. However, its low crystallization ability and slow crystallization rate [8] limit its wider applications in more fields. The addition of a nucleating agent was considered as one of the most effective ways to improve its crystallization properties. At present, the commonly used nucleating agents for polylactic acid mainly include inorganic nucleating agents, e.g., talc [9,10], clay [11], organic nucleating agents such as sorbitol [12] and ethylene bis(stearamide) [13] and polymer nucleating agents [14]. These nucleating agents have all been proven to increase the crystallinity or crystallization rate of PLA. However, they have a certain impact on the degradability of the polymer, i.e., it fails to achieve full degradation and affect the original biocompatibility of PLA to a certain extent. Therefore, nucleating agents with little or no negative effect on the degradability of polylactic acid are desirable. In this regard, the development of a nucleating agent based on a biomass system for the crystallization modification of polylactic acid has received research attention. This paper reports on several common biomass nucleating agents to regulate the crystallization of PLA for the construction of pure green material systems.

## 2. PLA

**Figure 1:**
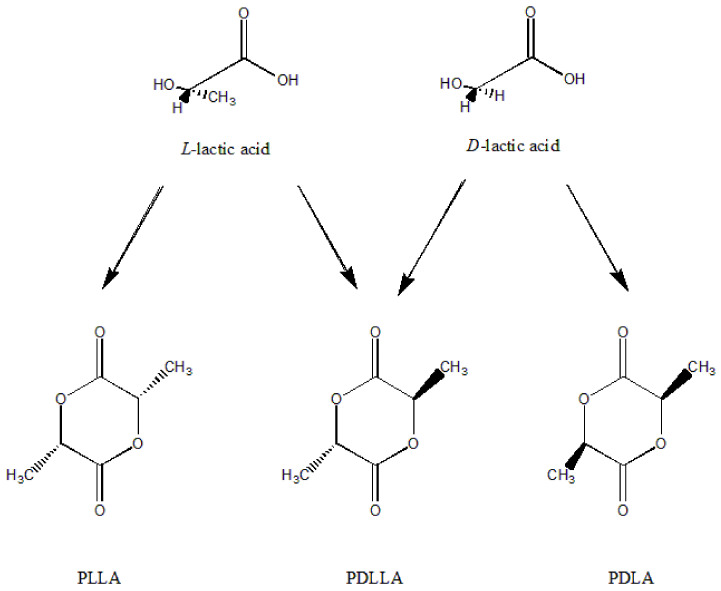
The structure of lactide and lactic acid.

PLA is a biopolymer synthesized from lactic acid [15]. Due to the presence of chiral carbon atoms, the conformation can be divided into two types: *L*- and *D*-. Therefore, PLA has three configurations: *D*-lactic acid (PDLA), *L*-lactic acid (PLLA) and *D*,*L*-lactic acid (PDLLA) (Figure 1). The first two are crystalline polymers, and PDLLA is an amorphous polymer. PLA is usually PLLA unless otherwise specified.

For PLLA-based polylactic acid, the content of PDLA has a significant effect on its crystallization. PLA with a PDLA content exceeding 7% basically will not crystallize [16].

**Figure 2:**
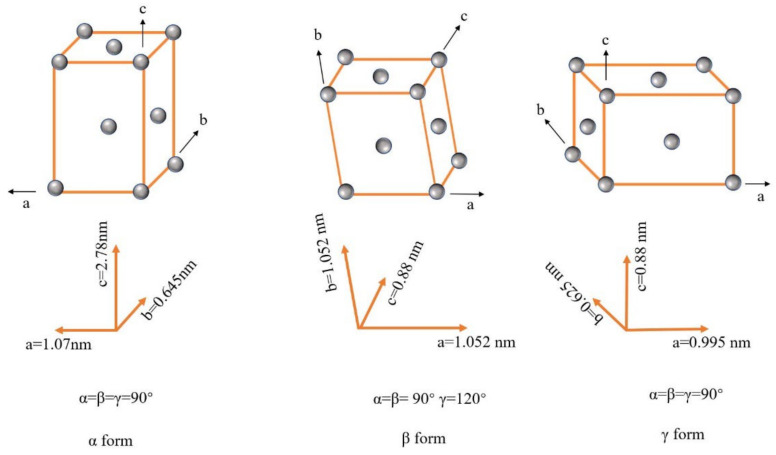
Crystal form model of PLA.

Depending on conditions such as the temperature during processing, PLA may take four different crystal forms, i.e., α, α′, β and γ [17,18,19]. The three crystal structure models of α, β and γ can be seen in Figure 2.

In the nonisothermal process, at temperatures below 100 °C, only the α′ crystal form exists. Its chain conformation and crystal system are similar to those of the α crystal form but more disordered and looser. When the temperature is further increased, the α′ crystal form begins to gradually transform into the α form. At a temperature higher than 120 °C, it is almost completely transformed into the α crystal form. This is the most common and thermodynamically stable one. The β crystal form can be obtained at a higher stretching rate and stretching temperature. It also has better impact properties and heat resistance. The γ crystal form is obtained by epitaxial crystallization on hexamethylbenzene [20]. Table 1 summarizes some properties of PLA with various crystal forms.

## 3. Biomass and Biomass Nucleating Agents

In a broad sense, biomass materials can be divided into plant-based biomass material, animal-based biomass material and microbial-based biomass material. Biomass is considered as one of the most promising renewable and sustainable resources due to its abundance and wide availability. It plays an important role in solving the depletion of petroleum resources and alleviating the environmental crisis caused by traditional petroleum-based polymer materials. In a narrow sense, biomass mainly refers to lignocellulosic material, such as woody plants, grasses, lianas and their processing residues and wastes as raw materials through high-tech means such as physical, chemical and biological approaches.

As mentioned earlier, most of the nucleating agents for PLA reported so far are traditionally petroleum-based. Despite their ability to enhance the crystallization of PLA, they give rise to the deterioration of properties including biocompatibility. Therefore, it is not suitable for biomedical fields with high biosafety requirements. In view of these considerations, the development of a nucleating agent belonging to the same biomass system as PLA has become a research focus.

Examples of reported biomass nucleating agents include: lignocellulosic materials (hemicellulose, lignin and cellulose) in plant cell walls [26,27,28]; natural fiber and wood powder from wood [29,30]; chitin and chitosan in the protective epidermis of crustaceans and the cell walls of some fungi [31,32,33]; and starch and its natural product, cyclodextrin [34,35]. The addition of these biomass materials to PLA was found not only to maintain the original biodegradability of PLA but also to improve its crystallization performance. The crystallization data of some biomass nucleating agents are shown in Table 2.

Most biomass nucleating agents are similar to traditional petroleum-based nucleating agents. The crystallization ability of PLA was improved by promoting the heterogeneous nucleation of PLA. When the small molecular biomass nucleating agent is added, it is dispersed in the whole PLA matrix. The site at the beginning of the crystallization of PLA increases, and the nucleation density increases. Therefore, the heterogeneous nucleation of PLA can occur at the position of a small molecule biomass nucleating agent and reduce the nucleation time. Furthermore, the crystallization rate of PLA is accelerated, and the crystallinity of PLA is improved. The crystallization parameters of some biomass nucleating agents in isothermal and nonisothermal processes can be seen in Table 3.

### 3.1. Cellulose

Cellulose, as the most abundant renewable compound available from nature, is widely found in plants, algae and some bacteria [27]. It is a macromolecular polysaccharide composed of glucose and is the main component of plant cell walls.

Shazleen and coworkers [37] prepared PLA/cellulose nanofiber (CNF) nanocomposites by melting and blending. Under the load of 1–3 wt.% CNF, no caking or clustering of CNF was observed, and a uniform dispersion in the PLA matrix and strong adhesion between the CNF and PLA matrix were shown. Therefore, the small molecular nucleating agent CNF can act as a heterogeneous nucleation site in the PLA matrix, thus improving the crystallization rate and crystallinity of PLA. In the nonisothermal crystallization process, the crystallization temperature increased from 106 °C to 115 °C at a higher CNF loading, with the cold crystallization peak moved to the low temperature, and the cold crystallization enthalpy decreased. A higher CNF loading of 3 wt.% led to the disappearance of the cold crystallization peak and an obvious increase in crystallinity from 2.3% to 44.2%. Even a higher loading of CNF resulted in decreased crystallinity with a minimum of 26.1% observed at 6 wt.% CNF loading. The crystallization of PLA was found to be very slow in the isothermal crystallization process, and the crystallization peak could hardly be observed on the DSC curve. In contrast, after the addition of CNF, an obvious crystallization peak appeared, and when adding 3 wt.% CNF, the crystallization peak was the sharpest. At the same time, by observing the isothermal curves at different temperatures, it was found that the crystallization of PLA was fastest at 100 °C, and the half-crystallization time was greatly reduced from 92.72 min to 1.40 min. When CNF loadings were higher than 3 wt.%, the crystallization behavior was similar to that of the nonisothermal crystallization process, and the crystallinity was observed to fall back. This phenomenon is probably because CNF with high loading is more prone to aggregation, reducing the mobility of the PLA chain and thus inhibiting crystallization.

Although CNF is conducive to nucleation when added to PLA due to its high specific surface area, due to its polarity, natural CNF has poor compatibility with and poor dispersion in nonpolar PLA, which limits the maximum potential of CNF and other nanocelluloses as nucleating agents for PLA. If CNF can be well dispersed in PLA and its compatibility can be improved, the promotion of PLA crystallization will be further improved. To this end, different methods have been developed to improve the compatibility of CNF and PLA, such as grafting plasticizers onto CNF or coating CNF with lignin to improve the dispersity of CNF in PLA. These efforts all contributed to an enhanced nucleation effect of CNF. Clarkson and coworkers [44] reported the uniform dispersion of a very small amount of cellulose nanocrystals (CNCS) and cellulose nanofibers (CNF) into PLA by using polyethylene glycol (PEG) as the plasticizer and studies on isothermal crystallization of PLA at 90–130 °C. They found that the crystallization of PLA was the fastest at 100 °C, and the semicrystallization time of PLA decreased from 1.66 min to 1.20 min and 1.16 min, respectively, after the addition of 0.05 wt.% CNC and CNF. Secondly, a theoretical calculation found that the nucleation coefficient and folding surface energy of PLA decreased from 8.69 × 10^5^ and 217.3 to 5.51 × 10^5^, 6.97 × 10^5^ and 141.0 and 176.1, respectively. The changes in the nucleation coefficient and folding surface energy before and after the addition of CNCS and CNF indicate that the addition of CNCS and CNF can improve the crystallization capacity of PLA by reducing the surface energy required for the stable nucleation of PLA. In a very small concentration, the crystallization capacity of PLA can be significantly improved. CNCS and CNF can thus be regarded as an effective nucleating agent for PLA. By comparing the surface-free energy of CNCS and CNF, it was found that CNCS had a higher surface-free energy than CNF (Table 4, which was consistent with its stronger nucleation ability. Therefore, CNCS could be considered a stronger nucleating agent. The specific surface area and surface-free energy of the nucleating agent were also important factors affecting the crystallization ability of PLA.

Gupta and coworkers [38] added lignin-coated cellulose nanocrystals (L-CNCs) to PLA. Due to the presence of lignin, a lignin layer can be formed on the surface of the cellulose nanocrystals (CNCS) through the interaction of electrostatic forces and van der Waals forces, preventing the reaggregation of CNCS during processing and improving the interface interaction between CNCS and PLA. L-CNCS can be used as an effective nucleating agent for PLA. The crystallization behavior of PLA was studied by DSC. It was found that adding 0.3 wt.% L-CNCs had the best nucleation effect on PLA. Compared with pure PLA, the crystallinity of the composites significantly increased from 6% to 40%, which was due to the reduction in the hydrogen bonds between the CNCS particles due to the presence of lignin. The CNCS were better dispersed in the PLA and provided more nucleation sites, thus improving the crystallization rate of PLA and promoting the growth of the PLA crystal. However, the continued addition of L-CNCs would lead to a decrease in crystallinity, which may be due to excessive agglomeration of the L-CNCs, leading to poor dispersion in the PLA. It was also found by calculation that the nucleation coefficient and folding surface energy of PLA decreased from 3.72 × 10^5^ and 8.25 × 10^−2^ to 3.32 × 10^5^ and 7.33 × 10^−2^, respectively, with the addition of 0.3 wt.% L-CNCs and increased with the further increasing addition of L-CNCs. It is also indicated that L-CNCs can be used as an effective nucleating agent for PLA at a low dosage. On the basis of this work, Boruvka and coworkers [36] adopted a two-step predispersion process combining an ultrasonic solution and mechanical agitation to better disperse the L-CNCs in PLA and prepared a PLA/L-CNC composite by melt blending. The results showed that the nucleation density increased, the thermal stability was enhanced, and the cold crystallization temperature decreased from 106 °C to 101 °C for the PLA. The crystallinity was also improved. This significant promoting effect on crystallization indicated that L-CNCs can be used as an effective nucleating agent for PLA.

**Figure 3:**
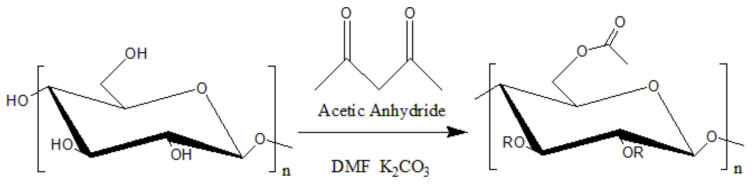
Acetylation modification of MFLC.

Yetis and coworkers [49] believed that the poor compatibility between cellulose and PLA affected the ability of cellulose as a nucleating agent. Therefore, the surface of microfibrillated cellulose (MFLC) was first acetylated to reduce the polarity of the MFLC and improve its compatibility with nonpolar PLA by increasing the interface adhesion between the MFLC and the nonpolar polymer matrix, enabling its even dispersion in the PLA (Figure 3). Then, the Ac-MFLC/PLA biocomposite film was prepared. Through a DSC test, it was found that in the nonisothermal crystallization process, the crystallinity of the composite film increased from 23.3% of pure PLA to 28.9% owing to the nucleation effect of the Ac-MFLC of a certain content (0.5–4 wt.%). Higher loading up to 5 wt.% resulted in a lower crystallinity (23.6%) than other biocomposites, which is due to the aggregation of microfibers and thus the reduction in nucleation sites [26].

### 3.2. Hemicellulose

Hemicellulose accounts for 25–35% of lignocellulosic materials and is a heteropolysaccharide, containing many different 5-carbon sugar and 6-carbon sugar monomers, such as xylan, mannose, galactose, arabinose. Monosaccharides are connected through covalent bonds and ester bonds and are also one of the components of plant cell walls [50].

Fundador [41] and coworkers studied the effects of different xylan esters on the crystallization properties of PLLA. In the nonisothermal crystallization process, xylan propionate and xylan butyrate were found to reduce the crystallization temperature of PLLA from 125 °C to 97 °C, respectively, and to increase the melting enthalpy from 17 J/g to 33 J/g and 34 J/g, respectively. During isothermal crystallization, the semicrystallization time decreased from 10.2 min to 6.9 min and 7.1 min, respectively. The change in spherulites during the isothermal crystallization was observed using a polarizing microscope. Compared with PLA without xylan ester, the spherulites were more dense and smaller with the addition of xylan ester because xylan ester acted as the nucleation site, thus improving the crystallization rate. Fundador attributed this nucleation effect to the similar spacing between xylan ester and one of the lattice constants of PLLA. The lattice constants of the α crystals of PLLA are a = 1.07 nm, b = 0.65 nm and c = 2.78 nm, while the spacing values of xylan propionate and butyrate are 1.28 nm and 1.48 nm, respectively. These are close to the lattice constants of the α crystals of PLLA along the a-plane and the lattice constants along the b-plane. This matching relationship induced the nucleation effect on PLLA. These results indicate that xylan propionate and xylan butyrate greatly improved the crystallization performance of PLLA and can be used as effective nucleating agents.

Another separate study found [51] that in the nonisothermal process, at a cooling rate of 20 °C/min, no crystallization peak appeared in the pure PDLA, while a significant crystallization peak appeared in the PDLA after the addition of 1 wt.% xylan ester, indicating that xylan ester can act as nucleating agent at a higher cooling rate and improve the crystallization capacity of PLA under rapid cooling conditions. In the isothermal crystallization process, compared with pure PDLA, the half-crystallization time was shortened from 2.5 min to 0.4 min. The results showed that adding xylan ester significantly improved the crystallization.

### 3.3. Lignin

**Figure 4:**
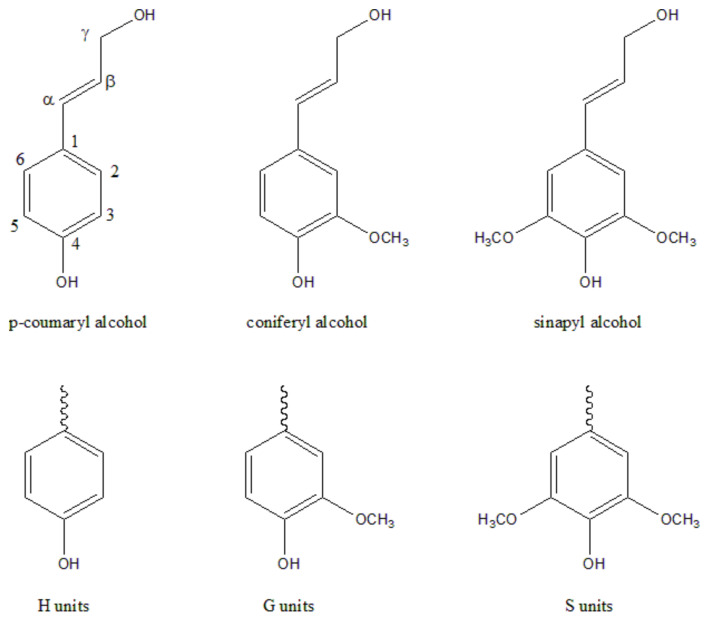
Main structural units of lignin.

**Figure 5:**
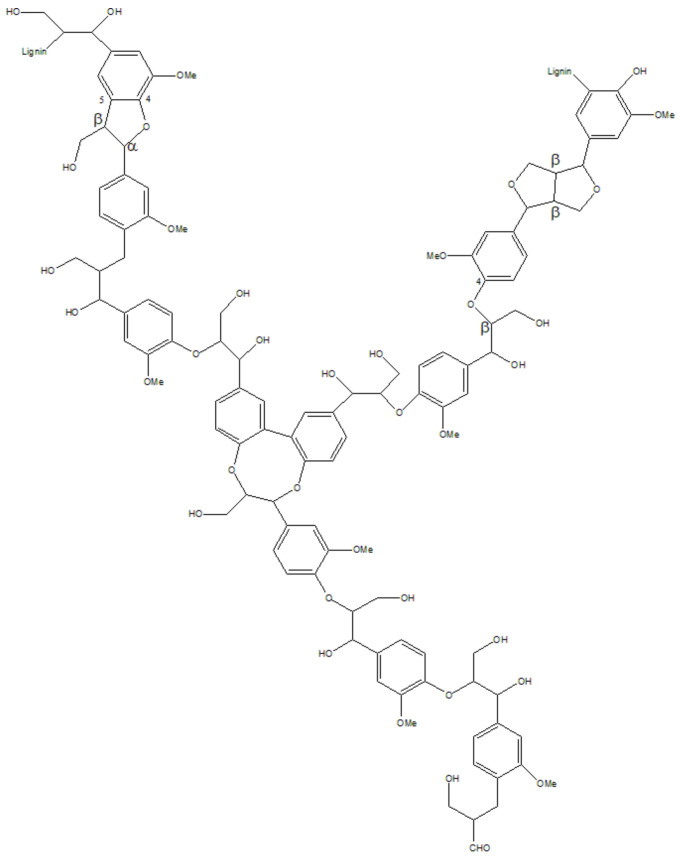
Lignin structure model.

Lignin, the second most abundant renewable resource after cellulose [52], is a biopolymer with a three-dimensional network structure formed by three phenylpropane units (p-coumaryl alcohol, coniferyl alcohol and sinapyl alcohol) (Figure 4) interconnected by an ether bond and carbon–carbon single bond [53]. Its structural model is shown in Figure 5. This biopolymer is characteristic of the structure and active groups of many aromatic rings. More than 50 million tons of lignin are produced each year, of which 98% is used for combustion power generation, and only 2% is used for fillers, dispersants and adhesives [54,55,56]. Lignin is almost insoluble under neutral and acidic conditions and has good solubility in glycol and a high pH (above 10) in aqueous solutions [57].

Kovalcik and coworkers [58] confirmed the potential of industrial lignin without any treatment as a nucleating agent of PLA. After the addition of 3 wt.% sulfate lignin (KL) and organic solvent lignin (OL), the crystallization peak of PLA moved towards a high temperature and became sharper during the cooling process; no cold crystallization peak appeared during the heating process. It indicated that the crystallization capacity of PLA was improved by adding lignin and that the crystallization can be perfected in the cooling process. Therefore, no cold crystallization peak appeared in the second heating. The crystallinity of pure PLA was found to increase from 25.5% to 57.8% and 55.0%, respectively. Calculated using Fallon’s nucleation efficiency formula, the nucleation efficiency was 31% and 27%, respectively. Using a polarizing microscope, it was observed that compared with pure PLA, the spherulite density increased at the same ratio after lignin was added. This also indicated that both KO and OL promoted the crystallization of PLA. At the same time, obvious dark spots could be clearly observed under the polarizing microscope, which was caused by the accumulation of lignin.

**Figure 6:**
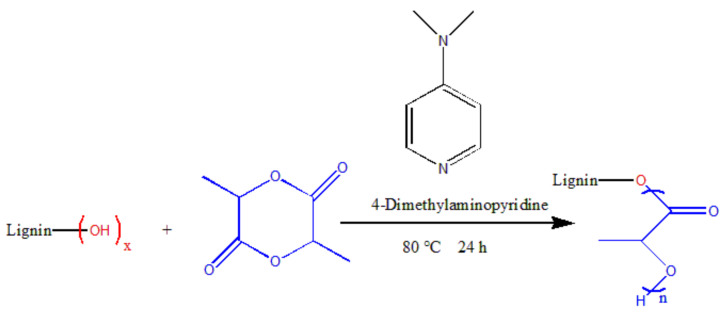
Synthesis of graft copolymers lignin-g-polylactic acid.

Although untreated lignin can be used as a nucleating agent for PLA, its effect of promoting PLA nucleation is weaker compared with modified lignin because the molecular structure of lignin contains a large number of hydroxyl groups and benzene rings. Hydrogen bonds are formed between hydroxyl groups, and π-π accumulation is formed between benzene rings. As a result, the compatibility of lignin and PLA is poor. In the PLA matrix, it is more likely to cluster together to form a cluster than to combine with PLA. In light of this, more research has been directed to the chemical or physical modification of lignin. Liu and coworkers [59] synthesized PLA–lignin graft copolymers with different chiral structures by incorporating lactide with different chiral structures into the lignin matrix (Figure 6). Then, this as-prepared LG-g-PDLA was added to PLA for composite film fabrication. The modification of lignin improved the interaction between the lignin and the PLA and thus improved their compatibility. It was found that lignin-g-PDLA showed good dispersion and compatibility in the composites. Compared with the other two (LG-g-PLLA, LG-g-PDLLA, LG-g-PDLA), it had a lower glass transition temperature, cold crystallization temperature and higher crystallization rate in the nonisothermal crystallization process. In the isothermal crystallization process, the crystallization peak became sharp at 1% LG-g-PDLA loading, and the half-crystallization time was shortened from 13 min to 5 min.

In most reports, microlignin was investigated with regard to its role as a nucleation agent. Large particles might adversely affect the interface adhesion between lignin and PLA [42]. A study where lignin nanoparticles (LNP) were added to PLA found that the crystallinity of the PLA increased from 15.0% to 22.5% at a 1% LNP loading in the nonisothermal crystallization process. The enhanced crystallization was attributed to the uniform dispersion of low-content LNP in the PLA and the nanoscale particle size of the LNP. LNP has a larger specific surface area, which is conducive to nucleation. However, at a higher LNP loading, the crystallinity began to decline, down to 17.4%, only slightly higher than that of pure PLA. The proposed reasons for this phenomenon are twofold, i.e., the aggregation of LNP at a higher content and the toughening effect rather than the nucleation of LNP [60].

Due to its complex and irregular structure, lignin is immiscible with most of the matrices. At present, the lignin used in most studies was obtained from separation and extraction by different physical or chemical methods. Therefore, the properties and relative molecular weight of the lignin obtained were quite different. The relative molecular weight of lignin is also one of the important factors affecting its compatibility with the matrix.

Singla [61] proved that the molecular weight of lignin had a great influence on the nucleation of PLA. The continued addition of low molecular weight lignin caused the cold crystallization temperature of PLA to rise at first and then decrease and for the crystallinity to first decrease and then increase. At a lignin content of 20%, the crystallization was enhanced compared with pure PLA, with crystallinity reaching 10.48%. This enhancement in crystallinity is due to the nucleation effect of low molecular weight lignin as a nucleating agent, and at lower lignin contents, the rigid groups of lignin hindered the movement of the PLA molecular chain, resulting in the decrease in crystallinity.

The increased loading of lignin led to enhanced nucleation, which exceeded the hindrance rigid groups on the PLA chain mobility and thus elevated the crystallinity. High molecular weight lignin will turn into a highly viscose tar in the heating process, and high viscosity lignin will affect the movement of the PLA chain segments. In addition, lignin will absorb energy during the heating process, reduce the energy transferred to the PLA and hinder the crystallization of PLA. Therefore, the addition of high molecular weight lignin will decrease the crystallinity of PLA.

### 3.4. Amino Acids

**Figure 7:**
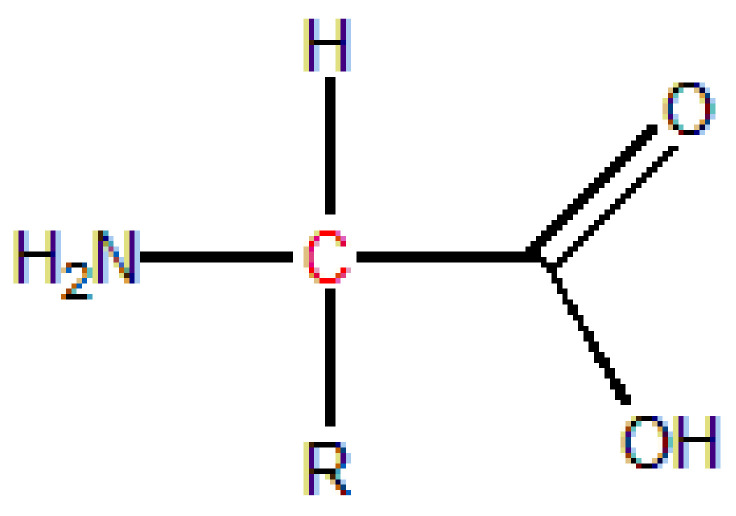
General structure formula of amino acids.

Amino acids are the basic unit of proteins, which do not only regulate the activity of biological macromolecules but also coordinate with a variety of metal ions. Amino acids have basic amino and acid carboxyl groups and contain chiral carbon atoms, so they have optical activity (Figure 7). The structure of amino acids is similar to that of PLA, so it can promote the formation of a crystal nucleus and can be used as the nucleating agent for PLA.

The structure of the R group in amino acids affects the crystallization and nucleation of PLA. The R group with a simple structure showed a small overall steric hindrance of the amino acids. Amino acids with structures similar to PLA have better miscibility with PLA. These two structure characteristics have a very significant promoting effect on the nucleation of PLA. In contrast, the nucleation effect is weak.

Marie and coworkers [62] added glycine, L-alanine, poly(glycine) and poly-DL-alanine into PLA to study their effects on the crystallization behavior of PLA. The results showed that these kinds of amino acids can be used as efficient biological nucleating agents for PLA, and the crystallinity of the composites can be greatly increased by adding a small amount (1.5%). Among them, poly(glycine) had the most significant effect, which can increase the crystallinity by 60.5%, which is equivalent to the talc powder (81.1%) used in the industry. This result is due to the fact that poly (glycine) has a simple structure, low steric hindrance and a high compatibility with PLA. Therefore, amino acids can be used as effective nucleating agents for PLA.

On the basis of the study by Marie, Wei [45] expanded his research and prepared a series of amino acid zinc salts and evaluated their role as nucleating agents for PLLA. At a cooling rate of 10 °C/min, the crystallization peak of PLA did not appear during the cooling process but appeared after the addition of zinc amino acid salt (1 wt.%). This indicates that all zinc amino acids can improve the crystallization capacity of PLLA. However, due to the differences in amino acid ligands and configuration, the nucleation ability was different, among which D-phenylalanine zinc salt was the most effective. When added to PLLA, the crystallization peak appeared at 130 °C, the melting enthalpy reached 48 J/g, the semi-crystallization time of PLA decreased from 35.2 min to 1.8 min, and the crystallinity increased to 56%.

In addition, it is proposed that the nucleation mechanism of PLLA induced by amino acid salt is based on the epitaxial nucleation mechanism of lattice matching. Amino zinc acid salt has a good lattice matching relationship between the PLLA crystal structure and its crystal structure (Table 5, so crystals can nucleate and grow on zinc amino acid salt through physical interaction. At the same time, the scanning electron microscopy found that the nucleation ability of amino zinc acid salt was also greatly related to the size and uniformity of particles. The smaller and more uniform the particles, the stronger the nucleation ability and the better the influence on the crystallization effect of the PLA.

### 3.5. Cyclodextrin

Cyclodextrin is a cyclic oligosaccharide composed of α-1,4-glucopyranoglucose. It is a slightly conical ring with a hydrophobic inner cavity and hydrophilic outer cavity, which has unique molecular container properties. It is widely used in biomedical fields [67].

Zhang and coworkers [34] first compounded low molecular weight PLA with α-cyclodextrin (PLA-IC) and then added it into PLA as a nucleating agent. It was found that at 118 °C, PLA needed 40 min to complete crystallization, while PLA/PLA-IC only needed 20 min, which reduced the time by half. The results showed that PLA-IC could significantly improve the crystallization rate of PLA. The semicrystallization time also significantly decreased from 11 min to 4.46 min. Due to the nucleation effect of PLA-IC, more heterogeneous nuclei were provided for the crystallization of PLA. It was observed using a polarizing microscope that the number of spherulites in the composite material greatly increased, and the grain size was much smaller at the same ratio. In addition, in order to further explore the nucleation mechanism of the PLA, the composite samples after quenching were observed using a polarizing microscope. It was found that the spherulites nucleated and grew around the PLA-IC particles, which again verified the nucleation effect of the PLA-IC particles.

**Figure 8:**
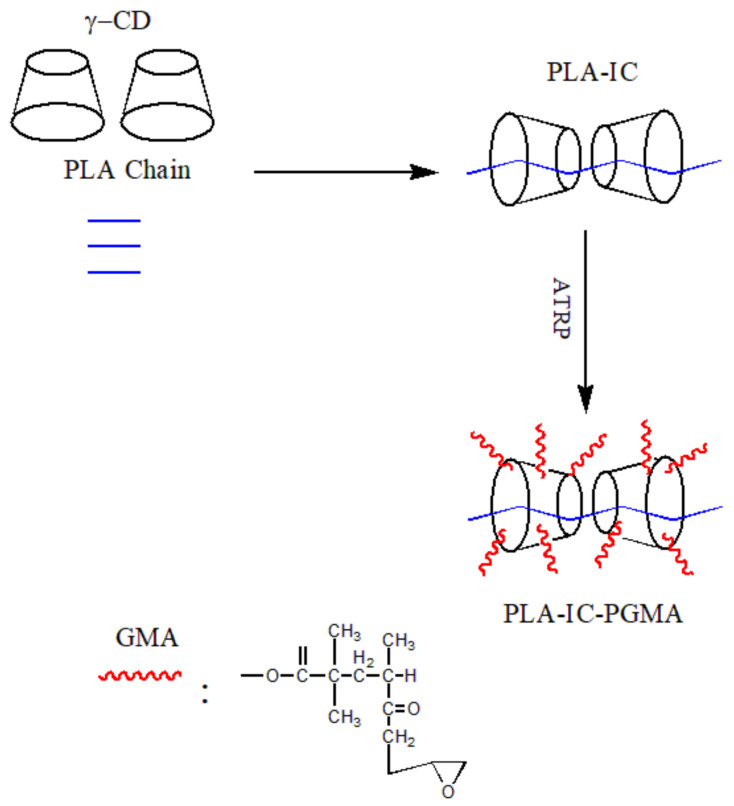
The synthetic route of polymers of PLA-IC-PGMA.

Li and co-workers [68] prepared a poly(lactic acid)/poly(lactic acid)-γ-cyclodextrin (IC)-poly(glycidyl methacrylate) (PGMA) composite by twin-screw extrusion (Figure 8). The DSC results showed that the melting temperature of the complex moved towards a low temperature, which is due to the formation of a hydrogen bond between the complex and PLA. It destroys the intermolecular interaction in PLA and enhances the movement ability of the PLA chain, thus, promoting its crystallization. In addition, the cold crystallization peak of the PLA disappeared during the second heating process, and the crystallinity increased from 5.17% of pure PLA to 30.73%. It shows that the complex acts as a heterogeneous nucleating agent and improves the crystallization ability of PLA.

### 3.6. Starch

Starch is a kind of semicrystalline polymer, which easily forms intermolecular and intramolecular hydrogen bonds in the crystalline region because of its hydroxyl group. In addition, its melting point is higher than the decomposition temperature. Therefore, starch is only used as a filler for thermoplastics with a low melting point. The field of application is very limited [69]. Moreover, due to the large amount of hydroxyl, it has poor compatibility with PLA which further affects its nucleation performance in PLA. Therefore, the modification of starch is an inevitable problem. At present, most studies use thermoplastic starch to reduce the size of starch in the dispersion of PLA by surface modification.

Dtttuam [47] studied the effect of untreated cassava starch on the crystallization behavior of PLA. It was found that with a low addition of cassava starch, the cold crystallization temperature of PLA decreased from 126 °C to 118 °C, the melting enthalpy increased, and the crystallinity increased from 3.3% to 6.2%. This is because cassava starch promotes the cold crystallization of PLA. At addition amounts higher than 5%, the crystallinity remained almost unchanged. At this time, a debonding phenomenon at the interfaces between the starch particles and the PLA matrix was observed using scanning electron microscopy. It showed that the less satisfied compatibility of untreated starch with PLA affected the promoting effect of starch as a nucleating agent on the crystallization of PLA.

Kang and coworkers [46] prepared chemically modified thermoplastic corn starch (CMPS)/PLA by twin-screw extrusion and studied its crystallization behavior. The results showed that the addition of CMPS could significantly improve the crystallinity and crystallization rate of PLA. The crystallinity of PLA increased from 2.4% to 30.1% when only 1% CMPS was added; the crystallization rate reached the maximum at 105 °C, and the half-crystallization time decreased from 24.7 min to 3.6 min. At the same time, it is pointed out that the nucleation rate is mainly controlled by free enthalpy and activation-free energy. The nucleation rate is mainly affected by free enthalpy at a high temperature region and greatly affected by activation-free energy at a low temperature. The pure PLA has a high enthalpy, so the nucleation rate is slow. When a nucleating agent is added, the enthalpy is reduced, and the nucleation at high temperature is promoted, and the nucleation rate is increased. Therefore, CMPS can be used as a fully biodegradable nucleating agent for PLA.

### 3.7. Wood Powder

Wu [43] studied the effect of wood meal (WF) as a nucleating agent on the crystallization behavior of PLA. During the nonisothermal crystallization process, the cold crystallization peak of PLA/WF composites shifted to a low temperature with the increase in WF content, and the cold crystallization peak became stronger. It showed that the cold crystallization of PLA was promoted. Compared with other components, the crystallinity of PLA increased the most when 4 wt.% WF was added. The crystallinity of PLA increased from 11.5% to 15.7%. In the process of isothermal crystallization, the sharpest crystallization peak was observed at a 4 wt.% WF addition at the same temperature. The semicrystallization time was shortened from 17.4 min to 2.8 min. The POM observation showed that there were more spherulites around the WF than in other regions after the WF was added. This is because the addition of WF could act as a nucleation site so that the spherulites could nucleate near it. Under the same POM ratio, it was found that at a 4% WF addition, the spherulite had the highest density and smallest size. The results showed that WF is an effective nucleating agent and that the nucleation effect was the best when the addition was 4 wt.%.

Silva [30] prepared cork polymer composites (CPC) and compared the nonisothermal crystallization behavior of PLA and composites at different heating rates using differential scanning calorimetry. It was found that the cold crystallization temperature and half crystallization time of the composites decreased, which indicated that cork could be used as a nucleating agent of PLA. In addition, by measuring the activation energy, it was found that the activation energy of PLA greatly decreased after cork was added, from 141.5 kJ/mol and 172.3 kJ/mol to 63.3 kJ/mol and 40.8 kJ/mol, respectively. The potential of cork as an effective nucleating agent for PLA was proved again.

### 3.8. Natural Plant Fiber

Borhan [70] studied the nonisothermal crystallization behavior of PLA/kenaf fiber (KF) composites at different cooling rates and different KF particle sizes. The results showed that when KF with a particle size between 80 μm and 106 μm was added into PLA, the spherulite size sharply decreased, the nucleation sites increased, and the fastest semicrystallization time was displayed. At higher cooling rates, the crystallization rate constantly increased, and the crystallinity was also improved. The activation energy was reduced to 161 kJ/mol compared with 173 kJ/mol of pure PLA, indicating that the addition of KF in this particle size range accelerated the crystallization of PLA. The results showed that KF with a particle size in the range of 80–106 μm could accelerate the crystallization of PLA and could be used as an effective nucleating agent of PLA.

Xu and coworkers [71] prepared a polylactic acid (PLA)/ramie fiber biocomposite and studied the effect of flow-induced morphology of the ramie fiber on PLA crystallization ability. The results showed that ramie fiber showed high nucleation activity to PLA due to its high flexibility and strong interface interaction between the fiber surface and PLA chain. With the increase in ramie fiber content (10–30 wt.%), the nucleation activity also increased. The average orientation parameter increased from 0.63 to 0.78, the cold crystallization temperature decreased from 92.6 °C to 88.3 °C, and the crystallinity increased from 9.8% to 16.8%.

## 4. Conclusions and Outlook

In recent years, there have been many studies on PLA biomass nucleating agents, including cellulose, hemicellulose, lignin, natural fiber and so on. These nucleating agents can effectively improve the crystallinity or crystallization rate of PLA and maintain the biodegradable characteristics of PLA, which plays a role in environmental protection. However, most biomass materials have poor compatibility with PLA due to abundant hydroxyl groups and more intermolecular and intramolecular hydrogen bonds.

Therefore, future research work could focus on the modification of biomass materials to improve their compatibility with PLA using physical or chemical modification approaches and to give full play to the maximum capacity of biomass materials as PLA nucleating agents. It is worth reminding that the selection of modification methods and modified substances should also proceed from biodegradability and biorenewability in order to maintain the biodegradability of PLA itself. Secondly, compared with the numerous types and extensive quantity of biomass materials, there is still few current studies on biomass materials as nucleating agents, so we can continue to excavate more biomass materials that can act as nucleating agents. Finally, most of the current studies are limited to the surface, without an in-depth excavation and exploration of the nucleation mechanism of biomass nucleating agents. Its applications in biomedicine and other aspects which especially need biosafety are rarely involved. These could be examples of potential areas that call for intensive attention.

## Figures and Tables

**Table 1:** Properties of different PLA crystal types.

Crystal Form	Crystal System	Chain Conformation	Cell Parameters					
			*a* (nm)	*b* (nm)	*c* (nm)	*α* (°)	*β* (°)	*γ* (°)
α [21]	Pseudo-orthorhombic	10_3_ helical	1.07	0.645	2.78	90	90	90
α [22]	Orthorhombic	10_3_ helical	1.05	0.61	2.88	90	90	90
β [23]	Orthorhombic	3_1_ helical	1.031	1.821	0.90	90	90	90
β [24]	Trigonal	3_1_ helical	1.052	1.052	0.88	90	90	120
γ [25]	Orthorhombic	3_1_ helical	0.995	0.625	0.88	90	90	90

**Table 2:** Crystallization data of biomass nucleating agents for different PLA.

Sample *	*T*_c_ (°C)	*T*_cc_ (°C)	*T*_m_ (°C)	*X*_c_ (%)	*t*_1/2_ ** (min)	*M*_w_ (g/mol)	*M*_n_ (g/mol)	PDI ***	Ref.
PLA 2500HP	94.6	95.6	176.6	25.3	-	10,812	6939	1.56	[12]
0.7%DS	110.2	-	177.5	51.4	-	10,812	6939	1.56	[12]
PLA 3251D	97	106	169	4.4	-	83,000	51,875	1.6	[36]
1%CNC	93	100	169	14.5	-	83,000	51,875	1.6	[36]
PLA 2003D	106.1	91.3	152.1	2.3	92.72	165,189	76,066	2.17	[37]
3%CNF	114.6	71.0	147.1	44.2	1.40	165,189	76,066	2.17	[37]
PLA 4043D	-	129.3	151.5	5.8	3.02	-	-	-	[38]
0.3%L-CNCs	-	112.5	146.6	40.0	1.36	-	-	-	[38]
PLA 4032D	96.7	-	168.2	36.9	7.90	188,000	98,000	1.92	[39]
2% Myo-inositol	137.3	-	166.2	40.0	2.69	188,000	98,000	1.92	[39]
PLA Shimadzu	-	115.5	169	45.8	16.8	186,340	121,000	1.54	[40]
1%Uracil	122.4	-	164.9	53.8	1.02	186,340	121,000	1.54	[40]
PLA Shimadzu	-	125	176	18.1	10.2	270,000	160,000	1.8	[41]
1%XylPr	-	97	171	35.6	6.9	270,000	160,000	1.8	[41]
PLA 3251D	-	100.3	169.3	15.0	-	-	-	-	[42]
1%LNP	-	100.3	168.1	22.5	-		-	-	[42]
PLA 4032D	-	114.6	160.7	11.5	17.4	-	-	-	[43]
4%WF	-	102.8	160.3	15.7	2.8	-	-	-	[43]

* CNC: cellulose nanocrystals; CNF: cellulose nanofibrils; L-CNCs: lignin-coated cellulose nanocrystals; DS: D-sorbitol; LNP: lignin nanoparticles; WF: wood floor. ** t_1/2_: Crystallization half-time. *** PDI = *M*_w_/*M*_n_.

**Table 3:** Crystallization parameters of different PLA biomass nucleating agents during isothermal and nonisothermal crystallization.

	Nonisothermal Crystallization				Isothermal Crystallization			
Sample *	*T*_c_ (°C)	*T*_cc_ (°C)	Heating/Cooling Rate (°C/min)	*X*_c_ (%)	*t*_1/2_ (min)	*K* ** (min^−n^)	*n* ***	Ref.
PLA	-	104	5	-	11	3.55 × 10^−3^	2.2	[34]
2%PLA-IC	94.5	-	5	-	4.46	3.00 × 10^−2^	2.1	[34]
PLA 2003D	106.1	91.3	2	2.3	92.72	2.83 × 10^−6^	2.74	[37]
3%CNF	114.6	71.0	2	44.2	1.40	2.45 × 10^−1^	3.11	[37]
PLA 4043D	-	129.3	10	5.8	3.02	1.53 × 10^−1^	1.5	[38]
0.3%L-CNCs	-	112.5	10	40.0	1.36	3.18 × 10^−1^	1.6	[38]
PLA Shimadzu	-	125	20	18.1	10.2	-	-	[41]
1%XylPr	-	97	20	35.6	6.9	-	-	[41]
PLA 4032D	-	114.6	10	11.5	17.4	2.3 × 10^−4^	2.8	[43]
4%WF	-	102.8	10	15.7	2.8	4.3 × 10^−2^	2.7	[43]
PLA 4032D	-	105.3	10	-	1.66	1.61 × 10^−1^	2.9	[44]
0.05 CNCS	-	88.0	10	-	1.16	4.36 × 10^−1^	2.7	[44]
PLA SUPLA	-	98	10	8.5	35.2	1.8 × 10^−3^	1.8	[45]
1%Zn(D-Phe)2	130	-	10	56	1.8	5.9 × 10^−1^	3.0	[45]
PLA 4032D	-	129	20	2.4	24.7	4.2 × 10^−4^	2.0	[46]
1%CMPS	-	113	20	30.1	3.6	2.4 × 10^−3^	2.8	[46]
PLA 2003D	-	125.9	10	3.3	4.6	5.43 × 10^−3^	2.9	[47]
5%Starch	-	118.2	10	6.2	3.7	4.5 × 10^−3^	3.5	[47]

* CNF: cellulose nanofibrils; CNCS: cellulose nanocrystals; L-CNCs: lignin-coated cellulose nanocrystals; XylPr: xylan propionate; PLA-IC: low molecular weight PLA in complex with α-cyclodextrin lignin nanoparticles; CMPS: chemically modified thermoplastic corn starch; WF: wood floor. ** *K*: crystallization rate constant. *** *n*: Avrami exponent.

**Table 4:** Surface energy for different materials at different temperatures.

Material	Temperature (°C)	Surface Energy (mJ/m^2^)
CNC [48]	30	46.5–66.9
	60	37.3–48.8
CNF [48]	30	44.3–52.5
	60	39–44.7

**Table 5:** Cell parameters of PLLA and zinc salts of amino acids.

Sample	Crystal System	Cell Parameters					
		*a* (nm)	*b* (nm)	*c* (nm)	*α* (°)	*β* (°)	*γ* (°)
PLLA [63]	Orthorhombic	1.066	0.616	2.888	90	90	90
Zn(L-Ala)_2_ [64]	Monoclinic	0.864	0.533	0.958	90	90.36	90
Zn(L-Pro)_2_ [65]	Monoclinic	0.961	0.565	1.049	90	91.63	90
Zn(L-Phe)_2_ [66]	Monoclinic	0.563	3.188	0.952	90	90.07	90

## Data Availability

Not applicable.
